# Zinc-salt assisted synthesis of three-dimensional oxygen and nitrogen co-doped hierarchical micro-meso porous carbon foam for supercapacitors

**DOI:** 10.1038/s41598-021-01151-3

**Published:** 2021-11-08

**Authors:** Hosein Banna Motejadded Emrooz, Ali Akbar Aghdaee, Mohammad Reza Rostami

**Affiliations:** Nanotechnology Department, School of Advanced Technologies, Iran University of Science and Technology, 16846 Narmak, Tehran Iran

**Keywords:** Chemistry, Energy science and technology, Materials science, Nanoscience and technology

## Abstract

Nitrogen and oxygen co-doped hierarchical micro-mesoporous carbon foams has been synthesized by pyrolyzation treatment of a preliminary foam containing melamine and formaldehyde as nitrogen, carbon and oxygen precursors and Zn(NO_3_)_2_. 6H_2_O and pluronic F127 as micro-meso pores generators. Several characterizations including thermal gravimetric analysis (TGA), X-ray diffraction (XRD) and Raman spectroscopy, FTIR and X-ray photoelectron spectroscopy, N_2_ adsorption–desorption, field emission scanning electron microscopy (FE-SEM) and transmission electron microscopy (TEM) were performed on the prepared foams. X-ray diffraction patterns, Raman spectra and N_2_ adsorption–desorption results confirmed that ZnO has pronounced effect on the graphitization of the prepared carbon foam. From X-ray diffraction, thermal gravimetric and N_2_ adsorption–desorption analysis results it was confirmed that the carbothermal reaction and the elimination of ZnO and also the elimination of pluronic F127 are the main factors for the induction of porosities in the foam structure. The presence of Zn(NO_3_)_2_. 6H_2_O and pluronic F127 in the initial composition of the preliminary foam results in the specific surface area as high as 1176 m^2^.g^−1^ and pore volume of 0.68 cm^3^.g^−1^. X-ray photoelectron and FTIR spectroscopy analyses results approved the presence of nitrogen (about 1.9 at %) in the form of pyridinic, graphitic and nitrogen oxide and oxygen (about 7.5 at. %) functional groups on the surface of the synthesized carbon foam. Electrochemistry analysis results including cyclic voltammetry (CV) and galvanostatic charge–discharge (GCD) and also electrochemical impedance spectroscopy (EIS) analysis illustrated the formation of an electric double layer supercapacitor with the capacitance as high as 137 Fg^−1^.

## Introduction

Environmental concerns from immoderate consumption of fossil fuels result in the attitude for new energy conversion and storage devices. In this attitude, the products of energy conversion and storage devices should be green and sustainable^1–3^. There are also ever-increasing demands for miniaturizing the portable electronic devices which in turn results in an urgent for high volumetric energy storage systems^4–7^. Both of these issues are meted in the electrochemical capacitors which are more known as supercapacitors and ultra-capacitors. Because of their unique properties such as excellent power density, long cyclic life and low charging time and also safety^8,9^, supercapacitors are widely used in the numerous applications such as portable electronics (power backups, memory systems), hybrid electric vehicles and also in the large industrial equipment^10,11^. Energy storage capability of the supercapacitors is due to the electrical double layer capacitance which comes from the electrostatic attraction between the charged electrode surface and the electrolyte ions and/or the pseudo-capacitance. The pseudo-capacitance of the capacitors is due to the oxidation–reduction (redox) or faradaic reactions. This charge transfer reaction is due to the presence of electroactive substances such as transition metal oxides or conductive polymers on the surface of the electrodes^12,13^.

Physicochemical characteristics of the electrode materials are the main determinative factors in the performance of supercapacitors. Specific capacitance as one of the most important characteristics of the supercapacitors mainly depends on the accessible surface area and the composition of the electrode materials. Micropores, particularly those with pore sizes less than 0.7 nm can increase the surface area of the electrode material which in turn can, potentially, enhance the capacitance, especially at low current densities. Presence of mesopores i. e pores with the sizes between 2 to 50 nm, and macro pores, pores with the sizes greater than 50 nm in the structure of the electrode are essential in the electrode performance. These porosities work as the diffusion path of the ions and, as a result, as a reducing factor for the equivalent system resistance. This, in turn, results in the higher power density of the supercapacitor. Therefore, hierarchical pore structure of the electrode materials is beneficial for its application as electrochemical capacitor^14,15^.

Availability, usually, high electrical conductivity and also large specific surface area has made the porous carbon materials as the first choice for supercapacitor electrode materials^16^. Considering the composition of the electrode material, introducing the heteroatoms such as oxygen, nitrogen, phosphorous and boron into the structure of the porous carbon can positively affect the capacitive behavior of the resulting supercapacitor. By increasing the electrical conductivity, chemical stability and also the electronegativity of the host material, heteroatoms can drastically affect both the electronic and crystalline structures of the porous carbon materials. These can even give rise to the pseudocapacitive reactions^12^.

Porous carbon materials can be synthesized applying several traditional methods, including hard and soft templating and carbonization- activation processes^17–20^. In the soft and hard templating techniques, carbon containing precursors are polymerized around a structure directing agent which can be another porous material such as porous silica (hard templating) or set of amphiphilic molecules (soft templating). After polymerization, the soft or hard templates are removed from the structure by chemical treatment of the product or by calcination^21–23^. The main advantages of the templating (soft or hard) techniques are the possibility of tuning the pore size and structure and also the possibility of the synthesis of ordered porous carbon materials. These characteristics confirm the good performance of the synthesized porous structure for batteries and supercapacitors. On the other hand, time consuming and expensive processes which come from the necessity of using, usually, expensive templates and also removing the templates after the synthesis procedures are drawbacks of these methods^17^. In carbonization- activation technique, usually, natural or waste organic materials were applied as the carbon sources. In this very common technique, the carbon precursors were etched by gaseous, liquid or solid substances at high temperatures, during which the porosities were induced in the structure. Cheap and sometimes gratuitous precursors with, mostly, worthy elements such as nitrogen, phosphorus, sulfur and oxygen are the main advantages of the carbonization- activation technique. On the other hand, to some extent, uncontrollable porosity formation processes and also low yield and the necessity of hazardous acids or alkaline compounds during the synthesis and post synthesis processes are the main drawbacks of this technique which affect its industrial application^4,24–27^.

Since the hierarchical structure of the porous carbon material is one of the main prerequisites for the proper performance of the prepared supercapacitor, selection of a synthesis technique in which the porous carbon products are naturally hierarchical, seems necessary. Salt templating technique for the synthesis of porous carbon introduced by Fechler et al., meets this condition. In this synthesis method, an inorganic non-carbonizable salt is mixed with a carbon precursor which is miscible with the salt throughout the reaction period. The carbon precursor and the salt can also be dissolved in a solvent. During condensation which can be taken place by freeze drying or heating, the solvent evaporates and the salt clusters or the heating product works as the templates for the porosity generation. Pyrolysis of the condensed product leads to the template evaporation and pore induction into the carbon material. In some cases, the products of the heating process can also act as a chemical agent for porosity induction. These products react with the carbon containing precursor and by etching process induce porosity in the carbon structure. With proper selection of the salt and the process parameters such as pyrolysis time and temperature and also, type and the ratio of the salt and the carbon precursor, one can tune the pore size and distribution^17,19,20,28^.

Hearin, we have introduced a new hierarchical three-dimensional nitrogen doped porous carbon by combining soft and salt templating and also chemical activation method. Zinc nitrate hexahydrate was selected as the structure directing and chemical activating agent and pluronic F127 as the soft template. Melamine–formaldehyde (also known as melamine resin) was selected as the carbon, oxygen and nitrogen precursors. After synthesis of the porous carbon, several characterization methods such as, X-ray diffraction, Fourier transformation infrared, Raman and X-ray photoelectron spectroscopy, N_2_ sorption, scanning, transmission electron microscopy and thermal gravimetric analysis have been conducted on the carbon products. Finally, the applicability of this carbon material was investigated in the electrode materials of the supercapacitors.

## Experimental section

### Materials

Hierarchical porous carbon particles were synthesized using melamine (Merck, Germany) and formaldehyde, 37% solution (Mojallali Co, Iran) as the carbon, oxygen and nitrogen precursors, ammonia, 25% aqueous solution (Mojallali Co, Iran) as the solvent agent of melamine, Zn(NO_3_)_2_.6H_2_O (Daejung Chemical Co, Korea) as the hard template and activating agent provider and pluronic F127 (Sigma Aldrich) as the structure directing agent. De-ionized water was used in all experiment. All chemicals were used as received without any purification.

### Synthesis of 3dimensional hierarchical porous carbon

In a typical synthesis, 2 g of melamine and 1.2 g pluronic F127 were added to the solution of 50 cm^3^ of formaldehyde, 37% and 10 cm^3^ of ammonia 25%. After complete dissolution of melamine and pluronic F127, 2 g Zn(NO_3_)_2_.6H_2_O was added to this solution and the mixture was stirred until dissolution of Zn(NO_3_)_2_.6H_2_O. The obtained solution was heated to 180 °C under vigorous stirring and the heating process continued until the appearance of a solid brown foam. This foam was nominated as CF-2. The same procedure was conducted for other specimens with 0, 4 and 6 g of Zn(NO_3_)_2_.6H_2_O as one of the nominated structure directing agent. The obtained solid foams were pyrolyzed for 3 h at the 1000 °C temperature under nitrogen inert atmosphere. The obtained carbon foams were nominated as CF-x-T which x denoted the amount of Zn(NO_3_)_2_.6H_2_O (0, 2, 4, 6 g) and T denoted as the pyrolyzing temperature. For evaluation of the pluronic F127 contribution in the porosity formation, a sample with the same procedure and without the addition of pluronic F127 has been prepared, named as CF-2w-1000. In order to determine the synthesis mechanism of the porous carbon, a specimen with the mentioned synthesis method was pyrolyzed for 3 h at 450 °C, CF-2-450.

### Characterization

Powder XRD analysis was performed on a BRUKER D8 ADVANCE diffractometer with Cu Kα (λ = 1.54 Å). N_2_ adsorption–desorption isotherms were performed on BELSORP mini II equipment at 77 K. Before beginning of any experiment, the specimens were degassed for 6 h at 150 °C. Teksan™^, Iran^ Raman spectrometer was applied for Raman Spectroscopy. FTIR spectra were obtained from Shimadzu 8400 s Spectrometer. X-ray photoelectron spectroscopy measurements were accomplished by an Al Ka source (XPS Spectrometer Kratos AXIS Supra). Field emission scanning and transmission electron microscopy (FE-SEM and TEM) analysis were conducted on 15 kV Mira3 TESCAN and 100 kV Philips TM 120, respectively. Thermal gravimetric analysis was performed under inert atmosphere using STA504 Bähr (Germany) up to 1000 °C.

### Electrochemical measurements

In order to carry out the electrochemical analysis, the working electrode was prepared as follows and coated on a graphite foam. About 4 mg of the synthesized porous carbon foam [CF-6–1000 (80 wt%)], 15 wt% carbon black and 5 wt% PTFE (polytetrafluoroethylene) as the binder were mixed in ethanol. The prepared slurry was then painted on the 1cm^2^ graphite foam and after drying overnight at 80 °C used as the working electrode. The electrochemical analysis was performed on VERASTAT Potentiostat & Galvanostat instrument by three electrode system with platinum as the counter and Ag–AgCl as the reference electrodes, respectively. The kinetical performance of the prepared electrode was investigated using electrochemical impedance spectroscopy (EIS) analysis in the frequency range of 0.01 to 10^5^ Hz. A solution of 6 M KOH was selected as the electrolyte for the galvanostatic charge–discharge and cyclic voltammetry analyses.

## Results and discussion

### Characteristics of the synthesized carbon foams

**Figure 1:**
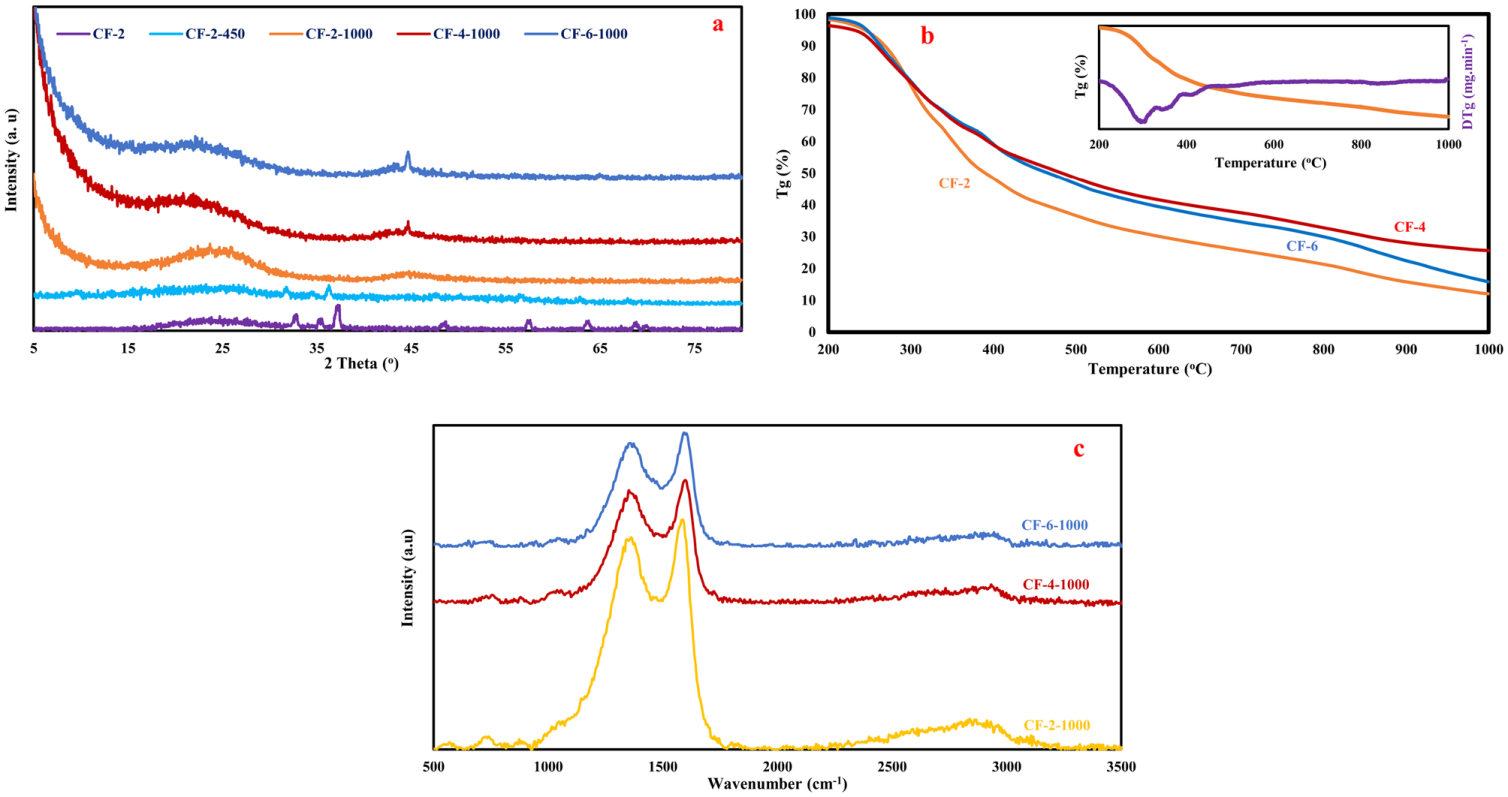
Structural development of the synthesized foam during thermal treatment (**a**) X-ray diffraction patterns of the synthesized foams, (**b**) Thermal gravimetric analysis results of the prepared foams with 2, 4 and 6 g of Zn(NO_3_)_2_.6H_2_O, (**c**) Raman Spectra of the carbon foams.

Synthesis mechanism of the prepared porous carbon foams can be inferred from the thermal gravimetric and powder X-ray diffraction analyses results of Fig. 1. According to the JCPSD number 001-00702551, the X-ray diffraction peaks at about 32°, 34°, 37°, 47°, 57°, 63°, 67°, 68°, 69° of the as prepared solid, (CF-2 in Fig. 1a), demonstrates the presence of hexagonal ZnO particles^29–31^ in the foam structure. This means that during the heating of the clear aqueous alkaline solution of melamine, formaldehyde, pluronic F127 and Zn(NO_3_)_2_.6H_2_O, in which the three dimensional solid brown structure was appeared, Zn(NO_3_)_2_ decomposes to ZnO^32^. The crystallite size of ZnO particles was calculated using Scherrer’s equation. Calculations revealed that the average crystallite size of these particles is about 13–15 nm. The lack of the characteristic peaks of melamine in the X-ray diffraction pattern of the as prepared foam along with the wide shoulder at about 25° confirm the reaction of melamine with formaldehyde and the formation of amorphous melamine–formaldehyde resin in the as prepared foam^33–36^. Decomposition of Zn(NO_3_)_2_ to ZnO particles, by the following chemical reaction, can be accompanied by the formation of carbon foam^32,37^:

$$Zn{({NO}_{3})}_{2}.6{H}_{2}O+{\varvec{C}}{\varvec{a}}{\varvec{r}}{\varvec{b}}{\varvec{o}}{\varvec{n}}\boldsymbol{ }{\varvec{P}}{\varvec{r}}{\varvec{e}}{\varvec{c}}{\varvec{u}}{\varvec{r}}{\varvec{s}}{\varvec{o}}{\varvec{r}}={C}_{Foam}+{NO}_{x}+{N}_{2}+{CO}_{2}+{H}_{2}O+ZnO$$

This reaction can take place due to the high oxidizing effect of Zn(NO_3_)_2_^32^. Due to the stability of the melamine–formaldehyde resin, this reaction is not the case in the present work. Therefore, based on the X-ray diffraction results and the brown color of the prepared foam, it can be concluded that Zn(NO_3_)_2_.6H_2_O is not so strong to convert melamine–formaldehyde resin to carbon particles. During the pyrolysis of the as-prepared solid brown foam to 450 °C, (CF-2-450) some of the characteristic peaks of zinc oxide disappeared, the intensity of other peaks decreased and their characteristic peaks shifted to the lower angles. Thermal gravimetric analysis results of CF-2, CF-4 and CF-6 presented in Fig. 1b, shows three weight loss regions which are approximately the same in all three specimens. In the first region, from the ambient temperature up to about 250 °C, the remained water evaporates from the solid structure. The sharp weight loss from 250 to 450 °C can be attributed to the removing of pluronic F127 and to some extent evaporation of ZnO nanoparticles. Evaporation of ZnO particles results in the decrease of the ZnO content of the foam. This is in agreement with the reduction in the intensity of ZnO characteristic X-ray diffraction peaks in the Fig. 1a. Based on the thermodynamic and kinetic characteristics of the carbothermal reduction of zinc oxide to zinc vapor, i. e. $ZnO+C={Zn}_{(v)}+CO$, it seems no possibility for etching of carbon elements in this range of temperature^38–40^. Continues weight loss with less intensity than that of 250–450 °C takes place in the temperature range of 450–1000 °C. This weight loss can be attributed to ZnO evaporation and etching of some of the carbons of the foam structure. DTG plot of CF-2 in the inset of Fig. 1b shows a tiny valley in the range of 800–900 °C. This valley was marked with a blue circle. It seems that this change in the slope of the weight loss is related to the beginning of the thermal reduction of carbon with ZnO particles (etching process). At the end of the thermal process all of ZnO particles have been eliminated from the foam structure and only turbostratic carbons remained which can be inferred from the X-ray diffraction results of CF-2-1000, CF-4-1000 and CF-6-1000 in Fig. 1a. Sharp peak at around 45° in CF-4-1000 and CF-6-1000 can be assigned to the (101) planes of graphite structure^41^. The graphitization extent of the synthesized carbons and the effect of zinc nitrate content of the initial mixture on the graphite formation can be inferred from their Raman spectra of Fig. 1c. The distinct peak at nearly 1356 cm^−1^ can be related to the disordered carbons with sp^3^ hybridization, named as D band and the peak at nearly 1634 cm^−1^ is attributed to the ordered graphitic carbons with sp^2^ hybridization, named as G band. The intensity of D and G band named as I_d_ and I_g_, respectively. The I_d_ to I_g_ ratios of the synthesized foams refers the graphitization extent of the synthesized carbons^24,42–44^. This ratio differs from 0.92 in CF-2-1000 to 0.90 in CF-4-1000 and 0.91 in CF-6-1000, respectively which are approximately the same. Therefore, and from the Raman results, there is no any considerable effect of zinc oxide content on the graphitization of the synthesized carbon particles. The broad band around 2800 cm^−1^ can be assigned to the formation of local graphene like structures^45,46^.

**Figure 2:**
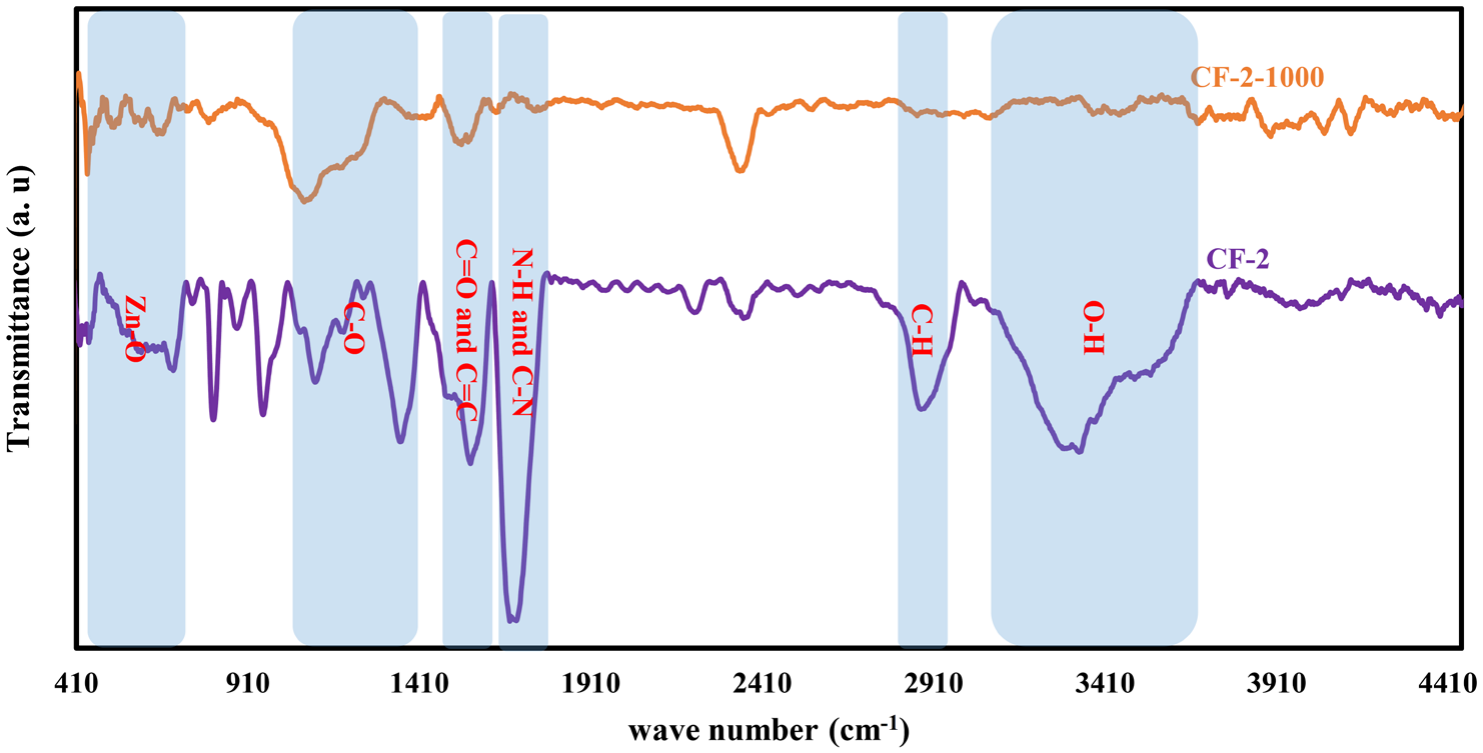
FTIR spectroscopy results of the synthesized (CF-2) and the pyrolyzed (CF-2-1000) carbon foam confirming the presence of C=C, C–N, C–O and C=O functional groups on the surface of the pyrolyzed foam.

Surface functionalities of the synthesized carbon foams can be inferred from the FTIR results of Fig. 2. The broad band from 476 to 726 cm^−1^ of CF-2 are related to Zn–O vibrational band^47^. From the FTIR result of the pyrolyzed carbon foam, CF-2-1000, it can be inferred that this band has been eliminated. The band ranging from 1000–1400 cm^−1^ is attributed to the C–O stretching^27,48^. For this range of wave number, pyrolysis treatment led to the broadening of the peak at 1100 cm^−1^ and elimination of the peak at 1350 cm^−1^. This means that pyrolysis treatment, to some extent, resulted in the elimination of oxygen element from the carbon structure. The peak at about 1560 cm^−1^ can be related to C=O carboxylic and C=C groups^48^ the intensity of which has been decreased during pyrolysis. The sharp peak for CF-2, located at about 1690 cm^−1^ which has been greatly decreased in CF-2-1000 is attributed to C–N and C–H groups^49^. Therefore, pyrolysis treatment results in the reduction of nitrogen element from the structure of the carbon foam, as well. The peak around 2900 cm^−1^ belongs to the C–H stretching^32,50^. The C–H functionality groups have also been eliminated after pyrolysis. The peak at around 3400 cm^−1^ which has been eliminated during the pyrolysis are attributed to the O–H hydroxyl group^50^. Therefore, and based on the FTIR results, it can be inferred that C–N, C–O and C=O functionality groups are present on the surface of the synthesized carbon foam.

**Figure 3:**
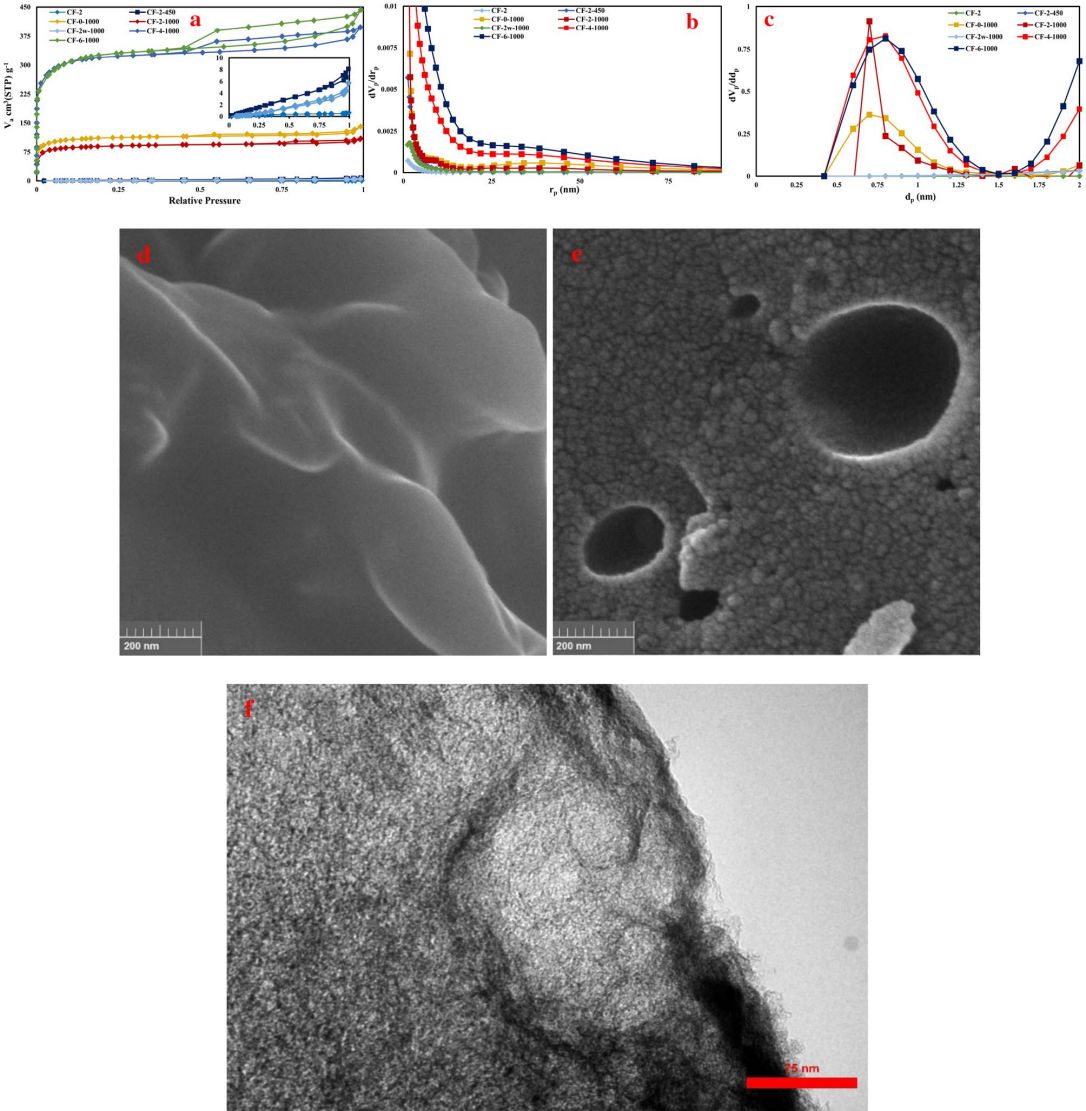
(**a**) N_2_ adsorption–desorption results of the synthesized carbon foams (**b**) BJH and (**c**) MP analysis of the carbon foams (**d**,**e**) Field emission scanning electron microscopy micrographs of CF-2 and CF-2-1000 (**e**) Transmission electron microscopy micrograph of CF-6-1000.

N_2_ adsorption–desorption analysis results of the as-synthesized (CF-2) and the pyrolyzed foams (CF-2-450, CF-2-1000, CF-2w-1000, CF-4-1000 and CF-6-1000) in concomitant with the field emission scanning electron microscopy micrographs of CF-2 and CF-2-1000 and transmission electron microscopy micrographs of CF-6-1000 are presented in Fig. 3a–f. Surface characteristics of the synthesized foams which have been extracted from the adsorption–desorption analysis results were presented in Table 1. From these data, the dominant effects of pyrolysis temperature, the addition of pluronic F127 and also the amount of Zn(NO_3_)_2_.6H_2_O on the specific surface area, pore volume and pore diameter is inferable. Before pyrolysis treatment, the synthesized foam, CF-2, has the surface area of about 0.75 m^2^g^−1^. The surface area of this foam will be increased to 4.6 m^2^g^−1^ after pyrolysis at 450 °C, CF-2-450 and to 334.12 m^2^g^−1^ after pyrolysis at 1000 °C, CF-2-1000. Considering the diffraction patterns of CF-2, CF-2-450 and CF-2-1000 in Fig. 1a, and the N_2_ adsorption–desorption results in Fig. 3a–c and Table 1, it can be concluded that the reduction in the ZnO content of the synthesized carbon foam during pyrolysis treatment at 450 °C has no any significant effect on the induction of porosity in the foam structure. Increasing the pyrolysis temperature to 1000 °C, results in the increase of the specific surface area from 4.6 to 334.12 m^2^g^−1^. The surface area and pore volume of the carbon foam with the addition of pluronic F127 and without the presence of Zn(NO_3_)_2_.6H_2_O, CF-0–1000, reached the amount of 414.66 m^2^g^−1^ and 0.22 cm^3^g^−1^ after pyrolyzation at 1000 °C, respectively. This is the highest amount of surface area that can be reached without the using of zinc nitrate hexahydrate. The importance of the presence of F127 in the initial composition of the carbon foam can be inferred from the comparison of the surface characteristics of carbon foams with and without F127 i. e CF-2-1000 and CF-2w-1000. The specific surface area and pore volume of CF-2-1000 is 334.12 m^2^g^−1^ and 0.17 cm^3^g^−1^, respectively while for CF-2w-1000, these are 1.02 m^2^g^−1^ and 0.01 cm^3^g^−1^, respectively. Therefore, in the present system, up to about 7.5 wt.% of zinc nitrate hexahydrate, this compound cannot lead to the significant amounts of porosities in the foam structure. On the other hand, CF-4-1000 and CF-6-1000 with about 14 and 20 wt.% of zinc nitrate hexahydrate have the specific surface area of 1175.8 and 1176.1 m^2^g^−1^, respectively. The pore volume of these two foams is 0.61 and 0.68 cm^3^g^−1^, respectively. This finding emphasizes that about 14 wt.% of zinc nitrate hexahydrate can significantly affect the surface characteristics of the synthesized foam. Similar I_d_ to I_g_ ratio of CF-2-1000, CF-4-1000 and CF-6-1000 (about 0.9) extracted from the Raman spectra of Fig. 1c, with the big difference in their specific surface area and pore volume, declares that ZnO has positive effect in the graphitization process. The larger the surface area is equivalent with the larger defect level. Therefore, approximately the same I_d_ to I_g_ ratio means higher value of graphitization ratio. The sharp X-ray diffraction peaks at about 45° for CF-4-1000 and CF-6-1000 porous carbons which belong to (101) planes of graphite (Fig. 1a), also emphasize that ZnO can encourage the graphitization process. Regarding the average crystallite size of ZnO particles, extracted from the XRD results of Fig. 1, 13–15 nm, it can be concluded that etching of the surface carbon atoms during the carbothermal reaction is the main role of ZnO in pore formation. For both CF-4-1000 and CF-6-1000, about 21 percent of the porosities are mesopores which means that the foam structure is to some extent hierarchical. The hierarchical characteristics of the synthesized carbon foam can accelerate the ionic transportation and this is helpful for its supercapacitor performance^8^. Field emission electron microscopy micrographs of the synthesized foam before (CF-2) and after the pyrolysis treatment (CF-2-1000) are presented in Fig. 3d,e. These micrographs clearly demonstrate on the porosity formation during the pyrolysis treatment which are in complete agreement with the results of Fig. 3a,b. The presence of micro porosities in the synthesized carbon foam structure ca be inferred from the transmission electron microscopy micrograph of CF-6-1000 in Fig. 3f.

**Table 1:** Surface characteristics of the synthesized carbon foams which have been extracted from the adsorption–desorption results.

	Total surface area (m^2^g^−1^)	Micropore surface area (m^2^g^−1^)	Total pore volume (cm^3^g^−1^)	Micropore volume (cm^3^g^−1^)	Mean pore diameter (nm)
CF-2	0.75	~ 0	~ 0	~ 0	4.9
CF-2-450	4.6	~ 0	0.01	~ 0	10.8
CF-0-1000	414.66	321.95	0.22	0.18	2.1
CF-2-1000	334.12	257.28	0.17	0.16	2.0
CF-2w-1000	1.02	~ 0	0.01	~ 0	33.8
CF-4-1000	1175.8	926.73	0.61	0.48	2.1
CF-6-1000	1176.1	892.95	0.68	0.54	2.3

**Figure 4:**
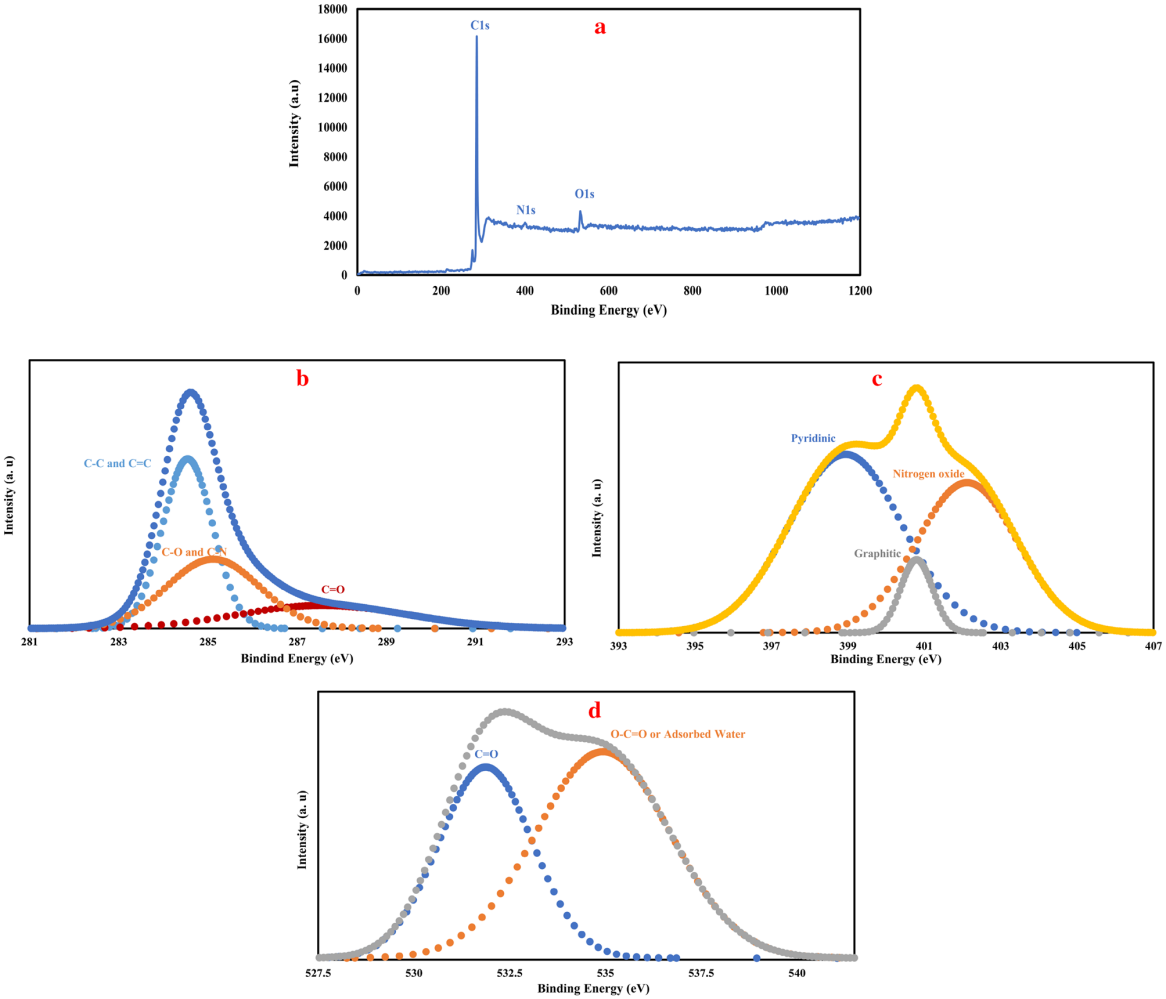
XPS spectra of the carbonized foam (CF-6-1000), (**a**) survey spectra showing the presence of C, N and O elements in the foam structure, (**b**–**d**) C1s, N1s and O1s deconvoluted spectra of the carbon foam.

From the X-ray photoelectron spectroscopy analysis results of the pyrolyzed carbon foam with the highest surface area (CF-6-1000) which has been presented in Fig. 4, it is possible to further insight in to the surface functional groups. The survey XPS diagram in Fig. 4a shows the presence of carbon, nitrogen and oxygen with the atomic percent of 90.6, 1.9 and 7.5, respectively. Deconvoluted spectra of C1s in Fig. 4b shows three peaks at 284.6, 285.2 and 287.7 eV. The prominent peak at 284.6 eV belongs to the graphitic carbons with C–C and C=C bindings ^5,51,52^. The peak at 285.2 eV is attributed to the C-N and C-O functional groups and finally the peak at 287.7 eV can be ascribed to C=O^53^. Deconvoluted N1s spectra of CF-6-1000 in Fig. 4c revealed that nitrogen atoms are present in three forms, pyridinic nitrogen at 398.7 eV, graphitic nitrogen at 401 eV and nitrogen oxide at 402.2 eV^3,25,42^. Finally, the O1s deconvoluted spectra of CF-6-1000 shows that can oxygen atoms are present in two forms, one at 531.6 eV which belongs to C=O functional groups and the other at 535.1 eV which is due to the carboxylic oxygen or adsorbed water^9,10,42,54^.

### Supercapacitor performance of the nitrogen and oxygen doped micro-meso porous carbon

**Figure 5:**
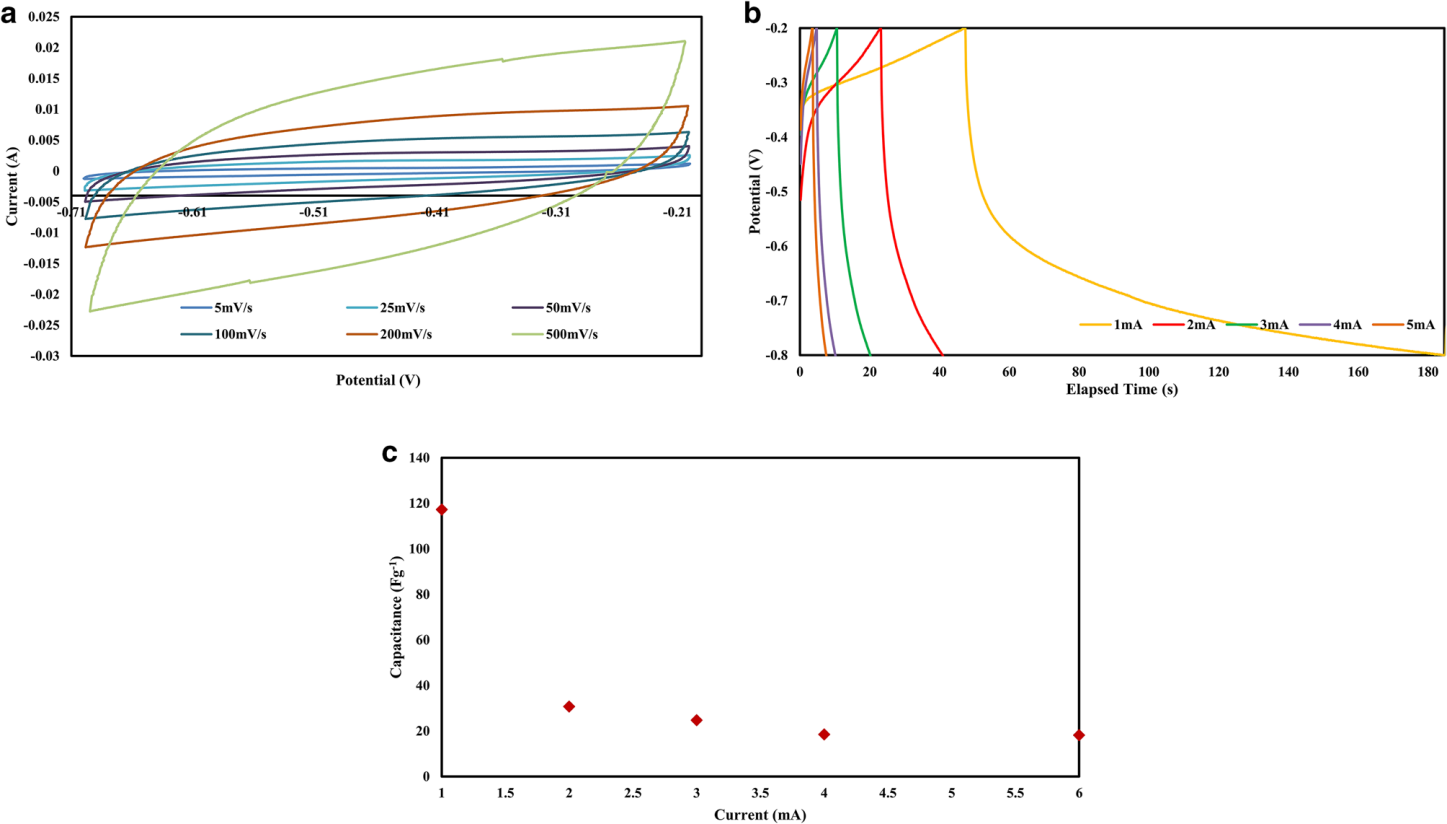
(**a**) Cyclic voltammetry (CV), (**b**) galvanostatic charge–discharge (GCD) of CF-6-1000, (**c**) the change in the capacitance of CF-6–1000 resulted from the change in current density.

Surface characteristics of the synthesized carbon foams including specific surface area, pore volume, pore size distribution and also surface functional groups can greatly affect their electrochemical performance. Based on these facts, CF-6-1000 with the highest specific surface area, pore volume, suitable ratio of micro-meso size porosities and surface nitrogen and oxygen functional groups has been selected for the electrochemical measurements. From the XPS results, the oxygen and nitrogen content of CF-6-1000 is about 7.5 and 1.9 at. %, respectively. Presence of these two heteroatoms on the surface can increase the hydrophilic characteristics of the synthesized carbon foam and improve the electrochemical performance^9,55^. The electrochemical performance of the nitrogen and oxygen doped hierarchical porous carbon including the cyclic voltammetry (CV), galvanostatic charge–discharge and gravimetric capacitance versus current density curves has been demonstrated in Fig. 5a–c. The quasi-rectangular shape of the cyclic voltammograms (CV) of CF-6-1000 almost in all the potential scan rates from 5 to 500 mVs^−1^, Fig. 5a, confirms the creation of a double-layer capacitor. The rectangular shape of the cyclic voltammograms results from the ions adsorption on the interface of the electrolyte and the working porous carbon electrode^56^. In higher scanning rates, the shape of the cyclic voltammetry curves, to some extents, deviates from the rectangle which can be related to the poor contact of the pore surfaces and the electrolyte during the charge and discharge process^57^. There is no any obvious peaks for redox reaction in the CV curves, but the high oxygen and nitrogen content of the activated carbon has a pronounced effect on the capacitance of the prepared capacitor^58^. The galvanostatic charge–discharge (GCD) curves the capacitance of the manufactured capacitor has been shown in Fig. 5b. As can be seen in the Fig. 5b, with increasing the current density, the discharge process shifts to the lower time. This may be due to the fast ionic transport with the increasing of the current density and also due to the internal resistance which arises from the hierarchical porous structure of the carbon foam^56^. From the GCD curves the capacitance at 1 A.g^−1^ scanning rate is about 117.3 F.g^−1^. This value for the capacitance of the prepared capacitor electrode is the result of relatively high surface area of the activated carbon (1176 m^2^g^−1^), high micropore surface area of about 893 m^2^g^−1^ and also the relatively high nitrogen and oxygen content of the porous activated carbon. In Table 2, the capacitance of some supercapacitors prepared from activated carbons has been compared. From this table it can be inferred that the ratio of the capacitance to the specific surface area of the activated carbon lies between 0.06 to 0.15, most of which are about 0.1. This emphasizes on the pronounced effect of the surface area, in comparison to the surface functionalities and the composition of the electrode materials, on the obtained capacitance. The capacitance to specific surface area ratio of the present work is about 0.1 which means that the obtained capacitance is in the expected value. With increasing the current density, the capacitance decreased to 30.7, 24.7, 18.5 and 18.2 F.g^−1^ at 2, 3, 4 and 6 A.g^−1^, respectively, Fig. 5c. This means that the rate capability of the prepared electrode is rather poor. High decreasing rates of the capacitance by increasing the current density may be attributed to the relatively high I_d_ to I_g_ ratio^56^ of the synthesized carbon foam in Fig. 1c. High I_d_ to I_g_ ratio implies that the ratio of amorphous carbons in the prepared activated carbon is relatively high. This corresponds to the low electrical conductivity of the synthesized active carbons^13^. The poor retention of the capacitance with increasing the current density can also be attributed to, relatively, low ratio of the mesopore to micropore surface area of the synthesized active carbon (⁓ 0.21). Poor mesopore porosities in comparison to the micropores, results in the poor electrolyte transfer and this, in turn, results in the poor retention of the capacitance with increasing the current density.

**Table 2:** Competition of the obtained capacitance from some activated carbons.

Specific surface area (m^2^.g^−1^)	Capacitance (F.g^−1^)	$\frac{{\varvec{C}}}{{\varvec{S}}{\varvec{S}}{\varvec{A}}}$	References
1041	**150**	**0.15**	^56^
2950	**320**	**0.10**	^62^
3300	**280**	**0.08**	^62^
2690	**260**	**0.09**	^62^
3420	**270**	**0.08**	^62^
2340	**280**	**0.12**	^37^
2230	**246**	**0.11**	^63^
2210	**239**	**0.11**	^63^
2760	**270**	**0.10**	^63^
1550	**95**	**0.06**	^59^
1688	**130**	**0.07**	^59^
1625	**110**	**0.06**	^59^
1580	**95**	**0.06**	^59^
1865	**180**	**0.09**	^60^
2236	**225**	**0.10**	^60^
2502	**265**	**0.10**	^60^
1176	**117**	**0.099**	**This work**

*C* capacitance, *SSA* specific surface area.

**Figure 6:**
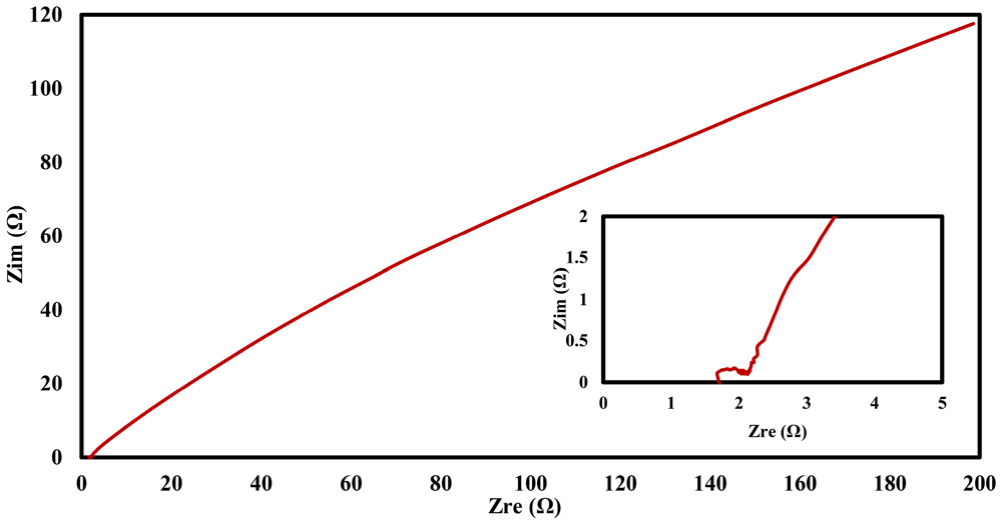
Nyquist plot of the supercapacitor electrode. The plot has been obtained from the electrochemical impedance spectroscopy (EIS) analysis results of electrode prepared from CF-6–1000, inset is the Nyquist plot at high frequency regions.

In order to study the ionic transport mechanism of the prepared electrode, electrochemical impedance spectroscopy (EIS) was carried out on the supercapacitor electrode, the result of which has been shown in the Nyquist plot in Fig. 6. A small semicircle can be detected in the high frequency region (the inset in Fig. 6). This semicircle followed by a 45° slope line in the intermediate frequencies. The series resistance which reflects the intrinsic resistance of the prepared electrode (Rp) and the electrolyte resistance in contact with the current collector (interface resistance) can be determined from the semicircle in the high frequency region. Rs can be determined from the left intercept of the Nyquist plot with the real x-axis which is about 1.7 Ω. Rp can be obtained from the right intercept of the Nyquist plot with the x-axis (about 2.1 Ω) and represents the internal or intrinsic resistance of the prepared electrode^59,60^. The obtained value for Rs and Rp is relatively high in comparison with some of the reported value^56,59,60^ and this is in line with the low retained capacitance in Fig. 5c. The intermediate region of frequency represents the resistive behavior of the ions penetrating into the electrode pores (diffuse layer resistance)^61^. This value can imply the level of the mesopores available for the transportation of the ions into the micropores. For the prepared electrode the diffuse layer resistance is greater than 190 Ω which is to some extent high in comparison to some reported value^56,59,60^. This high value emphasizes that the ratio of meso to micropores in the prepared electrode is relatively low.

## Conclusion

Nitrogen doped micro-mesoporous carbon foams has been synthesized using melamine and formaldehyde as nitrogen, oxygen and carbon precursors and Zn(NO_3_)_2_.6H_2_O and F127 as the templates for pores generation. The surface characteristics of the carbon foams synthesized from different amounts of the Zn(NO_3_)_2_.6H_2_O and pluronic F127 revealed the importance of these templates for the porosity formation. Pluronic F127 importance can be inferred from the specific surface area of carbon foams synthesized with (334.12 m^2^g^−1^) and without (1.02 m^2^g^−1^) using F127. The effectiveness of Zn(NO_3_)_2_.6H_2_O is obvious from the specific surface area changes of the synthesized foams resulted from the different amounts of zinc nitrate hexahydrate. It has been confirmed that during the pyrolysis treatments zinc oxide particles have positive role in graphitization of carbons. The electrochemistry results confirm the formation of electric double layer capacitor with the capacitance as high as 117.3 Fg^−1^.
